# A maple syrup extract alters lipid metabolism in obese type 2 diabetic model mice

**DOI:** 10.1186/s12986-019-0403-2

**Published:** 2019-12-04

**Authors:** Tsudoi Toyoda, Asuka Kamei, Tomoko Ishijima, Keiko Abe, Shinji Okada

**Affiliations:** 1 Graduate School of Agricultural and Life Sciences, The University of Tokyo, 1-1-1 Yayoi, Bunkyo-ku, Tokyo, 113-8657 Japan; 2 Group for Food Functionality Assessment, Kanagawa Institute of Industrial Science and Technology, 3-25-13 Tonomachi, Kawasaki-ku, Kawasaki, Kanagawa 210-0821 Japan

**Keywords:** Diabetes, Lipid metabolism, Maple syrup, Polyphenol

## Abstract

**Background:**

Some polyphenols are known to improve the symptoms of diabetes. In the present study, we investigated the effects of a polyphenol-rich extract of maple syrup (MSx) on a diabetic mouse model.

**Methods:**

KK-*A*^*y*^ mice were fed a normal or 0.05% MSx-supplemented diet for 42 days. Body weight, food intake, serum biochemical parameters, and fecal total bile acid were measured. Gene expression of liver and epididymal white adipose tissue (WAT) and cecal microbiota were analyzed. Data were analyzed with an unpaired two-tailed Student’s *t* test or Welch’s *t* test according to the results of the *F* test.

**Results:**

Serum low-density lipoprotein cholesterol levels were significantly reduced in mice that consumed MSx. Hepatic genes related to fatty acid degradation and cholesterol catabolism were upregulated in mice that consumed MSx. In contrast, the expression of genes related to lipid metabolism in WAT was unaffected by the intake of MSx. There were no significant differences between the two groups in terms of total bile acid level in the feces and the relative abundance of bacteria in the cecum.

**Conclusion:**

Our results primarily indicate that MSx can help alleviate one of the symptoms of dyslipidemia.

## Background

The increasing prevalence of diabetes is a global problem [1]. Diabetes mellitus is characterized by chronic hyperglycemia due to defects in insulin production and/or the response to insulin. Diabetes is classified as type 1 or 2 according to the underlying cause of these defects: type 1 results from the destruction of pancreatic β cells, whereas type 2 is due to environmental factors such as obesity, stress, and lack of exercise as well as genetics. Diabetes patients often have dyslipidemia, which is a risk factor for developing atherosclerotic cerebrovascular and cardiovascular diseases. Dyslipidemia is a pathology that promotes triglyceride degradation in adipose tissue and very low-density lipoprotein (VLDL) synthesis in the liver and suppresses VLDL catabolism in the blood due to insulin resistance. Some polyphenols are known to improve the symptoms of diabetes [2]. Brown alga polyphenols reduced nonfasting blood glucose levels in KK-*A*^*y*^ mice, a model for type 2 diabetes accompanied by obesity, and acacia polyphenols improved dyslipidemia and insulin resistance in KK-*A*^*y*^ mice [3, 4].

Maple syrup is a sweetener made from the sap of the sugar maple tree, *Acer saccharum*, which contains a large number of polyphenols [5]. A butanol extract of maple syrup polyphenols was shown to inhibit α-amylase and α-glucosidase activities in vitro [6]. Inhibiting these carbohydrate hydrogenases is expected to prevent an increase in blood glucose levels by delaying the absorption of carbohydrates. Additionally, the ethanol extract suppressed the production of nitric oxide and prostaglandin E2, a substance that induces inflammation, in RAW 264.7 cells activated by lipopolysaccharides [7]. In our previous study, we reported the effect of the ethanol extract on hepatic gene expression in C57BL/6 J mice fed a high-fat diet [8]. Maple sugar, which is produced by boiling and drying maple syrup, was shown to elevate blood glucose levels to a lesser degree than sucrose in Otsuka Long-Evans Tokushima Fatty rats, a type 2 diabetes model [9].

Based on the above reports, we speculated that maple syrup, particularly its polyphenol-enriched extract, could alleviate chronic hyperglycemia in individuals with diabetes. In the present study, we used KK-*A*^*y*^ mice and fed them an ethanol extract of maple syrup (MSx) containing 15.02% polyphenols as gallic acid equivalents for 42 days. Then, serum biochemical parameters related to nutrient metabolism, gene expression profiles of liver and epididymal white adipose tissue (WAT), and composition of intestinal bacteria were evaluated to determine the effects of MSx on nutrient metabolism, with the results demonstrating that MSx alters lipid metabolism.

## Methods

### MSx preparation

MSx prepared from Canadian maple syrup (Canada No. 2/Amber) by SiliCycle Inc. (Quebec, QC, Canada) was purchased by the Federation of Quebec Maple Syrup Producers (FPAQ; Longueuil, QC, Canada) [7]. Briefly, maple syrup was diluted with deionized water and was applied to an Amberlite XAD16 column (Sigma-Aldrich, St. Louis, MO, USA). The non-adsorbed fraction was eluted with deionized water and discarded. The adsorbed fraction was eluted with ethanol and evaporated in vacuo in a rotary evaporator. MSx refers to this dried fraction.

### Animals and diets

Male KK-*A*^*y*^ mice 4 weeks of age were purchased from CLEA Japan (Tokyo, Japan). The mice were individually housed in a room maintained at a temperature ranging from 21 to 23 °C with 50–70% relative humidity and a 12:12-h light/dark cycle. Normal and 0.05% MSx-supplemented diets were prepared based on the AIN-93G formula by Oriental Yeast Co. (Tokyo, Japan) (Table 1) [10]. After 4 days of acclimation, the mice were divided into two groups (*n* = 8 each) with approximately equal mean body weights. Mice were fed the normal or MSx diet for 42 days and were allowed free access to the diet and ultrapure water during this period. Body weight and food intake were measured every 2 days. Feces were collected on days 36–38 of the testing period and were stored at − 80 °C until use. After 16 h of food deprivation, mice were sacrificed under sodium pentobarbital anesthesia. Blood was collected from the heart chamber and centrifuged at 830×g for 10 min for serum isolation. The liver was treated with RNAlater (Thermo Fisher Scientific Inc., Waltham, MA, USA). Epididymal WAT and cecum contents were immediately frozen in liquid nitrogen. All samples were stored at − 80 °C until use.

**Table 1:** Diet composition

Ingredient	Normal diet	MSx-supplemented diet
	(g/kg diet)	
Corn starch	529.486	528.986
Casein	200.000	200.000
Sucrose	100.000	100.000
Soybean oil	70.000	70.000
Cellulose	50.000	50.000
AIN-93G mineral mix	35.000	35.000
AIN-93 vitamin mix	10.000	10.000
L-Cystine	3.000	3.000
Choline bitartrate	2.500	2.500
*tert*-Butylhydroquinone	0.014	0.014
MSx	–	0.500

Eight serum biochemical parameters, including glucose, glycated albumin, total cholesterol, low-density lipoprotein (LDL) cholesterol, high-density lipoprotein cholesterol (HDL), triglycerides, nonesterified fatty acids (NEFA), and total ketone bodies, were measured on a 7180 Clinical Analyzer (Hitachi High-Technologies, Tokyo, Japan) by Oriental Yeast Co. Serum insulin and tumor necrosis factor (TNF)-α levels were measured with a Mouse Insulin ELISA Kit (Morinaga Institute of Biological Science, Kanagawa, Japan) and a Mouse TNF-α Quantikine ELISA Kit (R&D Systems, Minneapolis, MN, USA), respectively. Homeostasis model assessment of insulin resistance (HOMA-IR) was calculated using the following formula:

HOMA-IR = insulin (ng/ml) × 26 μIU/ml × glucose (mg/dl).

Total cholesterols were isolated as total lipids from the liver according to the Folch method [11] and its levels were measured with the Cholesterol E-Test Wako (Wako Pure Chemical Industries, Osaka, Japan). The total bile acid level in the feces was measured with the Total Bile Acid Test Wako (Wako Pure Chemical Industries) as follows. Feces were lyophilized and ground with a mortar. A 5-fold volume of ethanol was added to the feces prior to heating at 70 °C for 1 h and centrifugation at 3500×g for 15 min, setting the supernatant aside. The same volume of ethanol was again added to the pellets and centrifuged as described above to set the supernatant aside. This procedure was repeated twice. The three supernatants were pooled for measurement.

Data were analyzed with the unpaired two-tailed Student’s *t* test or Welch’s *t* test according to the results of the *F* test. Differences were considered significant at *P* < 0.05.

### Analysis of gene expression in the liver and WAT using DNA microarray

Total RNA was isolated from the liver and WAT with TRIzol Reagent (Thermo Fisher Scientific Inc.) and was purified with an RNeasy Mini Kit and RNase-Free DNase Set (Qiagen, Venlo, the Netherlands). Total RNA concentration was measured on a spectrophotometer. RNA integrity was evaluated with an Agilent RNA 6000 Nano Kit and on an Agilent 2100 Bioanalyzer (Agilent Technologies, Santa Clara, CA, USA) and RNA Integrity Number was confirmed greater than 8.0. cRNA was prepared from purified 100 ng total RNA with the GeneChip 3′ IVT PLUS Reagent Kit and hybridized to a GeneChip Mouse Genome 430 2.0 Array (Thermo Fisher Scientific Inc.). The array was stained using a GeneChip Hybridization, Wash and Stain Kit and a GeneChip Fluidics Station 450 (Thermo Fisher Scientific Inc.). The fluorescence signals of the probes were scanned with a GeneChip Scanner 3000 7G (Thermo Fisher Scientific Inc.) and converted to an intensity value with Affymetrix GeneChip Command Console software (Thermo Fisher Scientific Inc.).

The intensity values of probe sets were normalized by the distribution-free weighted method in the case of liver and by the quantile normalization factor analysis for robust microarray summarization method in the case of WAT [12, 13]. Data were compared between the control and MSx groups using the rank products method [14]. Probe sets with a false discovery rate (FDR) < 0.001 in the liver were considered to show significant differences in expression. The threshold of FDR in the WAT was set to < 0.02 to obtain an approximately equal number of probe sets in the liver. Probe sets sorted as both up- and downregulated were excluded, and the genes for the remaining probe sets were treated as differentially expressed genes (DEGs). Normalization and between-group comparisons were performed with R v.3.2.2 [15] and Bioconductor v.3.1 [16]. DEGs were annotated according to biological process in Gene Ontology (GO) terms, which were enriched using the Database for Annotation, Visualization and Integrated Discovery v.6.7 (DAVID) [17]. Overrepresented GO terms were evaluated according to a modified Fisher’s exact test *p* value [18]. FDR was calculated from the *p* value using the Benjamini and Hochberg method [19]. GO terms in the liver and WAT with FDR < 0.01 were regarded as significantly enriched. The hierarchical structure of GO terms was determined with QuickGO [20]. DEGs annotated with GO terms related to lipid metabolism were mapped to metabolic pathways by Kyoto Encyclopedia of Genes and Genomes (KEGG) pathway analysis in the DAVID browser. The detailed functions of DEGs annotated with GO terms related to the immune system were searched with Universal Protein Resource Knowledgebase (UniProtKB) [21]. Ensemble IDs were assigned to DEGs with the annotation file Mouse 430 2 Annotations Release 35 [22].

### Quantification of expression level of hepatic genes related lipid metabolism by qRT-PCR

Total RNA (1 μg) isolated from the liver as described above was reverse transcribed with a SuperScript IV VILO Master Mix (Thermo Fisher Scientific Inc.). The synthesized cDNA (1 ng) was amplified in a 10-μL reaction volume with a PowerUp SYBR Green Master Mix (Thermo Fisher Scientific Inc.) on a CFX Connect Real-Time PCR Detection System with CFX Maestro 1.1 software (Bio-Rad Laboratories, Inc., Hercules, CA, USA) under the following conditions: 50 °C for 2 min, 95 °C for 2 min, and 40 cycles of 95 °C for 15 s and 60 °C for 1 min. Primers were designed with Primer3web v.4.1.0 (http://primer3.ut.ee/) and these sequences are shown in Additional file 1. The primer specificity was confirmed using dissociation curve. Expression level was measured using the calibration curve and that of each gene was normalized by that of a*ctin, beta* (*Actb*) whose expression showed the smallest differences between the two groups among 5 housekeeping genes. PCR reactions were performed with 3 technical replicates for each gene. DNA contamination was not detected.

### Analysis of cecal bacterial composition

The cecal microbiota was evaluated by the Central Institute for Experimental Animals (CIEA; Kanagawa, Japan) using a modified terminal restriction fragment length polymorphism (T-RFLP) method [23]. Briefly, genomic DNA was isolated from cecal contents and amplified with fluorophore-labeled primer sets designed for amplifying 16S rDNA. The amplified product was digested with a restriction enzyme, HpyCH4III, and analyzed on an ABI PRISM 310 Genetic Analyzer with GeneScan software (Thermo Fisher Scientific Inc.). Fragment length was assigned to an operational taxonomic unit (OTU), which is specific to bacteria in a microbiota database constructed by the CIEA. The OTU area was regarded as the number of bacteria.

## Results

### Physical and biochemical parameters

To examine the effect of MSx on diabetes, KK-*A*^*y*^ mice were fed a normal diet with or without 0.05% MSx for 42 days. Feces were collected on days 36–38. After fasting, mice were sacrificed, and serum samples were obtained. Values for physical and biochemical parameters are shown in Table 2. There were no significant differences between the two groups in terms of glycated albumin, glucose, insulin, or HOMA-IR levels. In contrast, LDL cholesterol levels were significantly lower in the MSx group than in the control group. These results indicate that chronic hyperglycemia, as a main symptom of diabetes, was unaffected, whereas cholesterol metabolism was altered by the intake of MSx.

**Table 2:** Physical and biochemical parameters of mice fed a normal or MSx-supplemented diet

	Control	MSx	*p*-value
Physical parameters			
Total food intake (g)	292.3 ± 7.1	293.3 ± 9.6	0.93
Final body weight (g)	36.9 ± 0.5	37.0 ± 0.5	0.95
Liver weight (g)	1.6 ± 0.0	1.6 ± 0.0	0.52
Serum biochemical parameters			
Glycated albumin (%)	8.3 ± 0.5	7.6 ± 0.5	0.40
Glucose (mg/dl)	77.0 ± 17.0	87.0 ± 11.0	0.62
Insulin (ng/ml)	2.1 ± 0.4	1.5 ± 0.2	0.21
HOMA-IR	10.0 ± 2.6	8.8 ± 2.1	0.71
Total cholesterol (mg/dl)	117.0 ± 5.0	114.0 ± 5.0	0.76
LDL cholesterol (mg/dl)	9.0 ± 1.0	6.0 ± 1.0^*^	0.01
HDL cholesterol (mg/dl)	64.0 ± 4.0	67.0 ± 3.0	0.62
Triglyceride (mg/dl)	98.0 ± 10.0	105.0 ± 14.0	0.73
NEFA (μmol/l)	619.0 ± 69.0	750.0 ± 99.0	0.30
Total ketone body (μmol/l)	452.0 ± 69.0	567.0 ± 61.0	0.23
TNF-α (pg/ml)	11.5 ± 2.5	7.4 ± 0.4	0.15
Liver biochemical parameters			
Total cholesterol (mg/g liver)	5.5 ± 0.3	6.0 ± 0.3	0.20
Fecal biochemical parameter			
Total bile acid (μmol/g feces)	1.6 ± 0.1	1.6 ± 0.1	0.90

Values represent the means ± SEMs of 8 mice. ^*^Significant difference at *p* < 0.05. *HOMA-IR* homeostasis model assessment of insulin resistance, *LDL* low-density lipoprotein, *HDL* high-density lipoprotein, *NEFA* Nonesterified fatty acid, *TNF* tumor necrosis factor

### Changes in the gene expression of the liver

To examine changes in cholesterol metabolism, we performed a hepatic transcriptome analysis using the DNA microarray technique. There were 272 and 265 DEGs that were up- and downregulated, respectively, in the MSx group compared to the expression levels in the control group (FDR < 0.001). A total of 537 DEGs were annotated with GO terms to categorize their function. DEGs were shown to be related to lipid metabolism (e.g., GO: 0006955 steroid metabolic process and GO: 0006954 fatty acid metabolic process) and the immune system (e.g., GO: 0006953 immune response, GO: 0009611 defense response, and GO: 0006694 response to wounding) (Table 3). These results suggest that lipid metabolism and immune function in the liver were altered by the intake of MSx. GO terms and annotated DEGs are listed in Additional file 2.

**Table 3:** GO terms annotated to genes differentially expressed in the liver

GO ID	GO term^a^	Number of genes
GO:0006955	Steroid metabolic process	18
GO:0006954	Fatty acid metabolic process	20
GO:0006953	Immune response	33
GO:0009611	Defense response	42
GO:0010033	└ Inflammatory response	29
GO:0055114	└ Acute inflammatory response	15
GO:0008202	└ Acute-phase response	9
GO:0006694	Response to wounding	33
GO:0051186	Oxidation reduction	53

*GO* Gene Ontology. ^a^ GO terms with FDR < 0.01

**Fig. 1:**
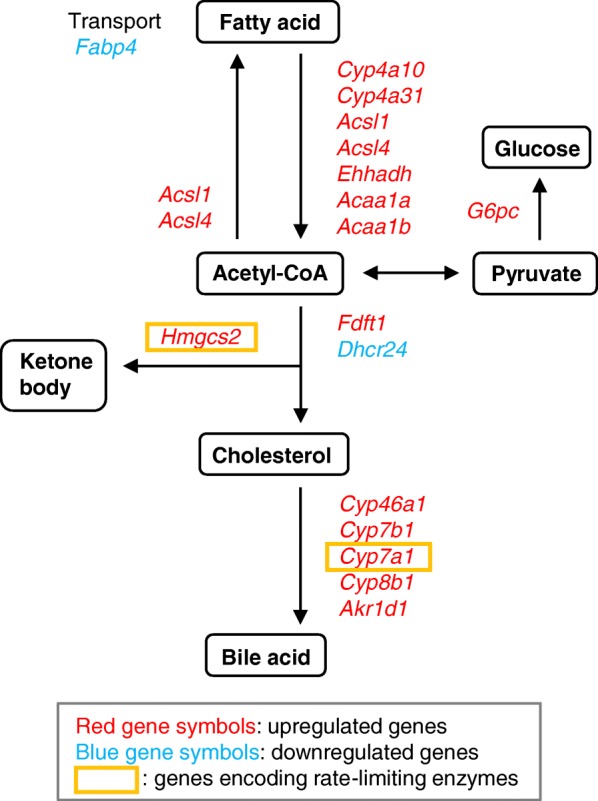
Metabolic pathway of DEGs related to lipid metabolism in the liver by DNA microarray analysis. DNA microarray analysis in the liver of mice fed a normal or MSx-supplemented diet was performed. DEGs annotated with GO terms of GO: 0006955 steroid metabolic process and GO: 0006954 fatty acid metabolic process are shown in an integrated metabolic pathway

To examine the effect of MSx on lipid metabolism, we mapped DEGs annotated with GO terms related to lipid metabolism in four KEGG pathways as follows: mmu00071 fatty acid metabolism, mmu00100 steroid biosynthesis, mmu00120 primary bile acid biosynthesis, and mmu00072 synthesis and degradation of ketone bodies. We integrated the KEGG pathways into a single metabolic pathway (Fig. 1). All DEGs mapped to the fatty acid degradation pathway were upregulated in the MSx compared to their expression in the control group. In the MSx group, *3-hydroxy-3-methylglutaryl-coenzyme A synthase 2* (*Hmgcs2*), encoding the rate-limiting enzyme in the ketogenesis pathway, was upregulated. These results suggest that fatty acid degradation is accelerated by the intake of MSx. All DEGs mapped to the cholesterol catabolic pathway were upregulated in the MSx group. Moreover, c*ytochrome P450, family 7, subfamily a, polypeptide 1* (*Cyp7a1*), encoding the rate-limiting enzyme, was included among the DEGs mapped to the pathway. This result suggests that cholesterol catabolism was stimulated by the intake of MSx.

**Fig. 2:**
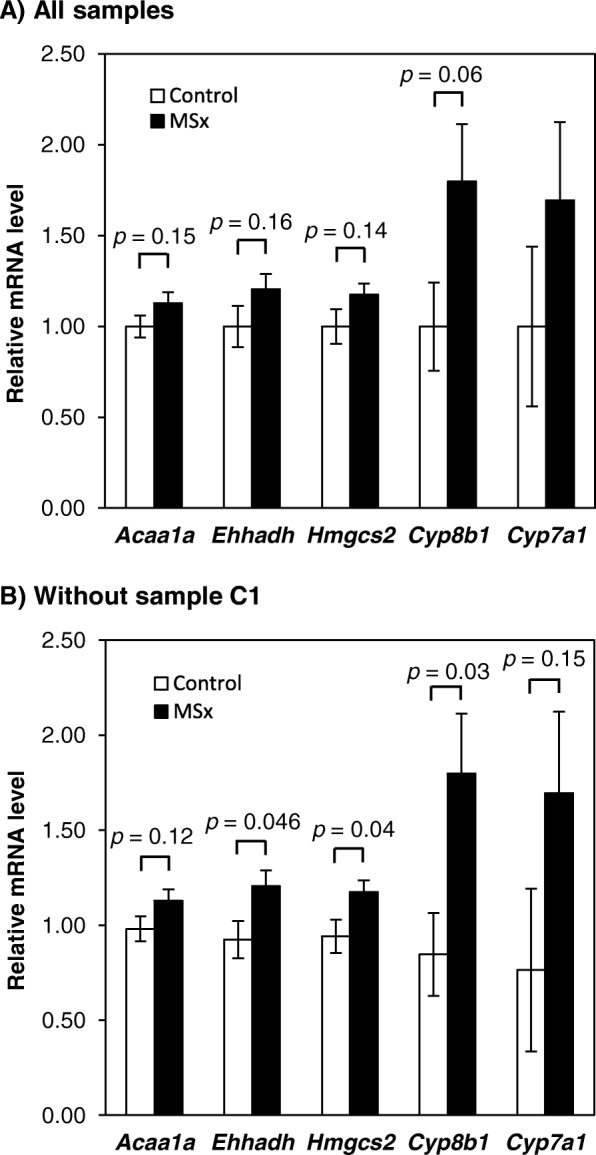
Expression levels of hepatic genes related to lipid metabolism by qRT-PCR analysis. qRT-PCR analysis in the liver of mice fed a normal or MSx-supplemented diet was performed. Values represent the means±SEMs of all samples (*n* = 8) (**a**) and the samples without C1 in the control group (**b**). The expression level of each gene was normalized against *Actb*

To determine the expression revels of 5 DEGs related to fatty acid β-oxidation, ketogenesis, and cholesterol catabolism, we performed qRT-PCR analysis. There were no significant differences between the two groups (Fig. 2a), although all of 5 DEGs were upregulated in the MSx group similarly to the results of DNA microarray analysis. Then, we reanalyzed the data without C1 sample because the expression levels of 5 DEGs in C1 ranked the top two and serum LDL cholesterol level in C1 was the lowest in the control group. As a result, 3 DEGs of *enoyl-Coenzyme A, hydratase/3-hydroxyacyl Coenzyme A dehydrogenase* (*Ehhadh*), *Hmgcs2*, and *cytochrome P450, family 8, subfamily b, polypeptide 1* (*Cyp8b1*) showed significant differences between the two groups (Fig. 2b).

Inflammation leads to the aggravation of diabetes symptoms by activating the immune system [24]. To examine the effect of MSx on the immune system, the biological function of the DEGs related to the immune system was examined using UniProtKB. Acute phase proteins encoded by *orosomucoid 1*/ *2*/ *3* (*Orm1*/ *2*/ *3*) and *serum amyloid A 1*/ *2*/ *4* (*Saa1*/ *2*/ *4*) are synthesized in the liver in response to inflammatory cytokines [25]. These DEGs were downregulated in the MSx group (Additional file 3). This result suggests that the inflammatory response was influenced by the intake of MSx.

### Changes in the gene expression of the WAT

The hepatic gene expression profile suggested that lipid catabolism increased with the intake of MSx. A gene expression analysis of WAT was performed to examine the effect of MSx on lipid metabolism in the tissue. There were 241 and 298 DEGs that were up- and downregulated, respectively, in the MSx group compared to the control group (FDR < 0.02). A total of 539 DEGs were annotated with GO terms. DEGs related to lipid metabolism were not distinctly presented (Table 4). These results suggest that MSx intake did not influence functions related to lipid metabolism in WAT. GO terms and annotated DEGs are listed in Additional file 4.

**Table 4:** GO terms annotated to genes differentially expressed in WAT

GO ID	GO term^a^	Number of genes
GO:0006955	Immune response	33
GO:0006952	Defense response	31
GO:0006954	└ Inflammatory response	24
GO:0009611	Response to wounding	30
GO:0042330	Taxis	15
GO:0006935	└ Chemotaxis	15
GO:0010033	Response to organic substance	33

*GO* Gene Ontology. ^a^ GO terms with FDR < 0.01

### Cecal microbiota

The gut microbiota is reported to affect host lipid metabolism [26]. Therefore, the effect of MSx intake on the gut microbiota in the cecum, an active site of fermentation by intestinal bacteria in rodents, was examined by T-RFLP analysis. Seven bacterial OTUs were estimated in each group (Table 5). There were no significant differences between the two groups in terms of the relative abundance of bacteria. These results indicate that MSx intake did not influence gut microbial composition.

**Table 5:** Bacterial composition of cecal contents in mice fed a normal or MSx-supplemented diet

Bacteria		OTU area (%)	
Phylum	Lower group	Control	MSx
*Bacteroidetes*	*Bacteroidales*	16.13 ± 1.58	19.00 ± 1.30
*Firmicutes*	*Lactobacillus*	11.80 ± 1.28	10.09 ± 1.28
	*Clostridiales*	47.89 ± 1.83	47.17 ± 3.21
	*Erysipelotrichaceae*	7.14 ± 1.91	6.76 ± 3.12
	Subtotal	66.84 ± 1.18	64.02 ± 1.01
Other	*Coriobacteriales*	1.83 ± 0.07	1.75 ± 0.11
	*Mucispirillum*	0.37 ± 0.08	0.31 ± 0.12
	*Parasutterella*	2.83 ± 0.29	2.45 ± 0.26
	Others	12.02 ± 0.53	12.48 ± 0.60
	Subtotal	17.04 ± 0.78	16.98 ± 0.74

Values represent the means±SEMs of 8 mice. *OTU* operational taxonomic unit

## Discussion

**Fig. 3:**
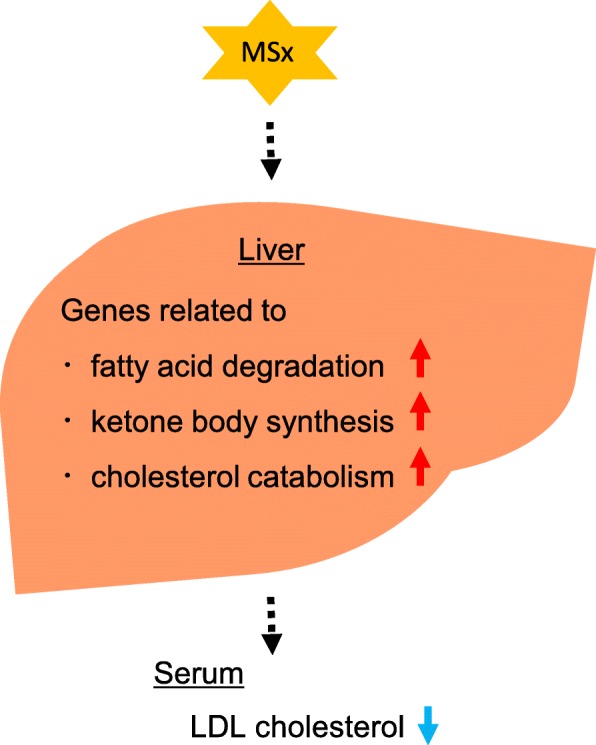
Effect of MSx intake in obese diabetic mice

According to the results of this study, dietary intake of MSx alters lipid metabolism (Fig. 3). The serum LDL cholesterol level and the hepatic gene expression profile suggested that MSx intake accelerates cholesterol catabolism and fatty acid degradation in the liver. Cholesterol synthesized in the liver is catabolized to bile acid or secreted into the blood as VLDL, which is then converted to LDL. This fact suggests that MSx intake causes cholesterol to be catabolized in the liver rather than being secreted into the blood. Ingestion of food components suppressing the absorption of bile acids from the intestine reduces LDL cholesterol level and promotes bile acid synthesis [27]. Thus, MSx may inhibit the absorption of bile acids; however, a difference between the groups in terms of the total bile acid level in feces was not observed. It is therefore likely that the LDL cholesterol-lowering effect of MSx was not caused by inhibiting the absorption of bile acids. Ketone bodies and cholesterol are synthesized from acetyl-CoA. Hepatic gene expression analysis using DNA microarray suggested that MSx intake promotes ketone body production and is supported by a slight elevation in serum total ketone body level (*p* = 0.23). Thus, MSx intake may cause the use of acetyl-CoA for ketogenesis rather than cholesterol synthesis, resulting in a decrease in serum LDL cholesterol levels. The enhanced ketone body production in diabetic patients is caused by the promoted utilization of fatty acids as an energy substrate due to the impairment of glucose utilization. However, there were no significant differences between the two groups with respect to the levels of serum glycated albumin, glucose, or insulin. This result indicates that MSx intake does not aggravate diabetic symptoms. Detailed research is needed to understand the mechanism of the underlying effect.

Various parameters (e.g. insulin, NEFA, and total ketone body in serum) in this study showed the large variance, which may be derived from an individual difference of KK-*A*^*y*^ mice. The results of qRT-PCR analysis were also affected by an individual difference, especially C1 in the control group. The effects of MSx intake on lipid metabolism of KK-*A*^*y*^ mice will be accurately revealed by increasing sample size.

In the context of diabetes, including in KK-*A*^*y*^ mice, the catabolism of lipoproteins such as VLDL and LDL is suppressed due to insulin resistance, resulting in the elevation of these lipoprotein cholesterol levels in blood [28], which is one of the pathologies of dyslipidemia. Our finding that serum LDL cholesterol level was lowered by MSx intake suggests that MSx can help alleviate one of the symptoms of dyslipidemia.

Using gene expression analysis of the liver and WAT, this study showed the possibility that MSx intake may affect the immune system. However, the results demonstrating the effect of MSx intake at the protein level could not be shown. Thus, further study is required to understand the effect of MSx intake on the immune system.

## Conclusion

The present study examined the effect of MSx intake in obese diabetic mice. The intake of MSx does not influence chronic hyperglycemia, but it reduces the LDL cholesterol level. Hepatic gene expression analysis suggested that the intake of MSx promotes cholesterol catabolism, although further studies are required to identify the detailed mechanism underlying the reduction in LDL cholesterol levels. Our findings provide evidence that MSx contributes to alleviating one of the symptoms of dyslipidemia.

## Supplementary information

**Additional file 1.** Primer information.

**Additional file 2.** GO terms and annotated DEGs in the liver. Up and down represent DEGs that were up- and downregulated, respectively, in mice fed MSx compared to the levels in controls. Fold change was calculated from the expression value of each probe normalized by the Affymetrix Micro Array Suite 5.0 method.

**Additional file 3.** DEGs related to the immune system in the liver. Down represents DEGs that were downregulated in mice fed MSx compared to the levels in controls.

**Additional file 4.** GO terms and annotated DEGs in WAT. Up and down represent DEGs that were up- and downregulated, respectively, in mice fed MSx compared to the levels in controls. Fold change was calculated from the expression value of each probe normalized by the Affymetrix Micro Array Suite 5.0 method.

## Data Availability

The datasets generated and/or analyzed during the current study are available in the Gene Expression Omnibus database at the National Center for Biotechnology Information, https://www.ncbi.nlm.nih.gov/geo/ (accession no: GSE112603 and GSE112687).

## Abbreviations

CIEA: Central Institute for Experimental Animals
DAVID: Database for Annotation, Visualization and Integrated Discovery
DEG: Differentially expressed gene
FDR: False discovery rate
GO: Gene Ontology
HDL: High-density lipoprotein
HOMA-IR: Homeostasis model assessment of insulin resistance
KEGG: Kyoto Encyclopedia of Genes and Genomes
LDL: Low-density lipoprotein
MSx: Maple syrup extract
NEFA: Nonesterified fatty acid
OTU: Operational taxonomic unit
TNF: Tumor necrosis factor
T-RFLP: terminal restriction fragment length polymorphism
UniProtKB: Universal Protein Resource Knowledgebase
VLDL: Very low-density lipoprotein
WAT: White adipose tissue
